# Supplementary material for: Restricted and non-essential redundancy of RNAi and piRNA pathways in mouse oocytes
Source: PLoS Genet. 2019 Dec 20;15(12):e1008261. doi: 10.1371/journal.pgen.1008261 (PMC6944382; doi:10.1371/journal.pgen.1008261)
Supplement: S1 Text — (DOCX) [file pgen.1008261.s001.docx]

**Title:**

Restricted and non-essential redundancy of RNAi and piRNA pathways in mouse oocytes

**Supporting Information**

**Running title:**

RNAi & piRNA redundancy

**Keywords:**

RNAi, Dicer, Mili, siRNA, piRNA, dsRNA, retrotransposon, oocyte

**Authors:**

Eliska Taborska^1^, Josef Pasulka^1^, Radek Malik^1^, Filip Horvat^1, 2^, Irena Jenickova^3^, Zoe Jelić Matošević^2^, Petr Svoboda^1+^

**Affiliations:**

^1^ Institute of Molecular Genetics of the Czech Academy of Sciences, Videnska 1083, 142 20 Prague 4, Czech Republic

^2^ Bioinformatics Group, Division of Molecular Biology, Department of Biology, Faculty of Science, University of Zagreb, 10000, Zagreb, Croatia

^3^ Czech Centre of Phenogenomics, Institute of Molecular Genetics of the Czech Academy of Sciences, Prumyslova 595, 252 50, Vestec, Czech Republic.

^+^ correspondence to: Petr Svoboda, Institute of Molecular Genetics ASCR, Videnska 1083, 142 20 Prague 4, Czech Republic, tel. # +420 241063147, e-mail: [svobodap@img.cas.cz](mailto:svobodap@img.cas.cz).

**Supplementary Methods**

**pSV40_RFP-T_ HA_mDicerSOM∆i2-i6_HR plasmid for homologous recombination**

A 5’ homology arm was generated by PCR amplification from plasmid containing HA-tagged Dicer exon 2 using primers pBS_Fwd1_seq & mDcr_e2-HA_Rev_PstICTGCAG (PstI restriction site was introduced at the 3’ end of the fragment through the primer). PCR fragment was cloned in pJET plasmid and sequenced. pBluescript (= pBS). A fragment containing Dicer cDNA sequence of exon 2 to exon 7 (DicerSOM fragment) was generated by PCR amplification from a plasmid containing Dicer cDNA sequence (pFastBACmf_DicerSOM) using primers mDcr_E2_Fwd_PstICTGCAG and mDcr_e7_Rev_ApaLIGTGCAC (PstI and ApaLI restriction sites were introduced at the 5’ and 3’end of the fragment respectively through the primers). PCR fragment was cloned in pJET plasmid and sequenced. A 3’ homology arm was generated by PCR amplification of Dicer intron 7 sequence using primers mDcr_i7_Fwd_PstICTGCAG and pHygro_Fwd_seq(R) (PstI restriction site was introduced at the 5’ end of the fragment with a primer). PCR fragment was cloned in pJET plasmid and sequenced.

To assemble the construct, the DicerSOM fragment was cleaved out from pJET using PstI and ApaLI enzymes, the 3’arm_HR fragment was cleaved out from pJET using ApaLI and ClaI enzymes and pBS was cleaved-opened by PstI and ClaI enzymes. The three fragments were ligated together. The 5’ arm_HR was cleaved out from pJET with BamHI and PstI. pBS_DicerSOM_3’arm_HR plasmid was also digested by BamHI and PstI. The 2 fragments were ligated together. Resulting construct pBS_HA_mDicerSOM∆i2-i6_HR was sequenced. HA_mDicerSOM∆i2-i6_HR template for homologous recombination was transferred into pSV40_RFPT backbone using BamHI and ClaI restriction sites.

**pSV40_RFP-T_ HA_mDicerSOM∆i2-i6_HR plasmid sequence**

LOCUS Exported 6692 bp ds-DNA circular SYN 18-APR-2019

DEFINITION pSV40_RFP-T_ HA_mDicerSOM∆i2-i6_HR plasmid

ACCESSION .

VERSION .

KEYWORDS .

SOURCE synthetic DNA construct

ORGANISM synthetic DNA construct

REFERENCE 1 (bases 1 to 6692)

AUTHORS .

TITLE Direct Submission

JOURNAL Exported Jun 1, 2019 from SnapGene 4.2.6

http://www.snapgene.com

FEATURES Location/Qualifiers

source 1..6692

/organism="synthetic DNA construct"

/mol_type="other DNA"

polyA_signal 468..689

/label=SV40 late polyA

/note="SV40 late poly(A) region"

misc_feature complement(698..2005)

/label=3-homology_arm

/note="mDicer 3'-homology arm (intron 7, long version)"

exon complement(2006..2174)

/label=exon_7

/note="mDicer exon 7"

exon complement(2175..2335)

/label=exon_6

/note="mDicer exon 6"

exon complement(2336..2470)

/label=exon_5

/note="mDicer exon 5"

exon complement(2471..2601)

/label=exon_4

/note="mDicer exon 4"

exon complement(2602..2764)

/label=exon_3

/note="mDicer exon 3"

exon complement(2765..2873)

/label=exon_2

/note="mDicer exon 2 (3' part)"

misc_signal complement(2873..2899)

/label=HA-tag

/note="HA tag"

exon complement(2906..2979)

/label=exon_2

/note="mDicer exon 2 (5' part)"

misc_feature 2980..3895

/label=5'-homology_arm

/note="mDicer 5'-homology arm (intron 1)"

CDS 4032..4892

/codon_start=1

/label=Amp_resistance

/note="ampicillin resistance"

/translation="MSIQHFRVALIPFFAAFCLPVFAHPETLVKVKDAEDQLGARVGYI

ELDLNSGKILESFRPEERFPMMSTFKVLLCGAVLSRIDAGQEQLGRRIHYSQNDLVEYS

PVTEKHLTDGMTVRELCSAAITMSDNTAANLLLTTIGGPKELTAFLHNMGDHVTRLDRW

EPELNEAIPNDERDTTMPVAMATTLRKLLTGELLTLASRQQLIDWMEADKVAGPLLRSA

LPAGWFIADKSGAGERGSRGIIAALGPDGKPSRIVVIYTTGSQATMDERNRQIAEIGAS

LIKHW"

rep_origin 5040..5707

/label=pUC origin

/note="pUC origin of replication"

misc_feature 5725..6143

/label=SV40 early enhancer/promoter

/note="SV40 early enhancer/promoter region"

intron 6204..6340

/label=Synthetic intron

/note="chimeric intron"

promoter 6384..6402

/label=T7 promoter

/note="T7 RNA polymerase promoter"

CDS join(6413..6692,1..454)

/label=RFP-T

/note="RFP-T"

ORIGIN

1 ggagagagtc accacatacg aagacggggg cgtgctgacc gctacccagg acaccagcct

61 ccaggacggc tgcctcatct acaacgtcaa gatcagaggg gtgaacttcc catccaacgg

121 ccctgtgatg cagaagaaaa cactcggctg ggaggccaac accgagatgc tgtaccccgc

181 tgacggcggc ctggaaggca gaaccgacat ggccctgaag ctcgtgggcg ggggccacct

241 gatctgcaac ttcaagacca catacagatc caagaaaccc gctaagaacc tcaagatgcc

301 cggcgtctac tatgtggacc acagactgga aagaatcaag gaggccgaca aagagaccta

361 cgtcgagcag cacgaggtgg ctgtggccag atactgcgac ctccctagca aactggggca

421 caaacttaat ggcatggacg agctgtacaa gtaacggccg cttcgagcag acatgataag

481 atacattgat gagtttggac aaaccacaac tagaatgcag tgaaaaaaat gctttatttg

541 tgaaatttgt gatgctattg ctttatttgt aaccattata agctgcaata aacaagttaa

601 caacaacaat tgcattcatt ttatgtttca ggttcagggg gaggtgtggg aggtttttta

661 aagcaagtaa aacctctaca aatgtggtaa aatcgatttt ccatggcagc tgagaatatt

721 gtaggagatc ttctagaaag attgtatgag atgtagaaac cgcactttag atgtagggtt

781 catcagcact acaactgcac catctggttg gtccctgcca gtgctgtgga gagctacggc

841 atgcagcagc gcaggccggc cttcctcttc agcaaagacc agagcgactt cccttcagct

901 gtactcatgc taactaatcc caagtcattc atacattttt ttttctatta taatcagtat

961 taaaatgaaa tggtaagatg tccaggtgac ttctggccag gccagcacga gaacttccct

1021 ctacaaaggc tggacaattt acaccaccgg gctggctctt atcttagagc tgacttgaaa

1081 aaaaaaatgt ggggcaatta ccagttttat atgcagtaaa gacagtgcgt tgaatatgga

1141 ctaagatttg taaggttaaa aaacccaaac aaggtaaggc atggtgacac acctttaatc

1201 ctggcactca ggagacagag gcaggtggac ctttgtgagt tagaggccag actagtctac

1261 attgtgagtt ccaggacaac cagagacaca cagtgagact ctctcaaaaa caattaaaaa

1321 aaaaaacaaa acaaaacaaa acaaaaaaaa aaacaaccta gacatggctg ttaaaaaatt

1381 aaatcaccac caagaagaat tctgagcctc atttaagagg gtgctgatat taaaactggc

1441 tgcaagcccc tagagtgctt tctggttgtc aagcatcagt gctggcggga ccactggagg

1501 gacctgtcag actcagtccc ttctgcggag ccctcctcac cgactcagca ggccaggaat

1561 gggtccccta aatttccctt caaccaacca gctgaggcgg caggtgttgt ggggctgctg

1621 cacgcgtgga accaactcaa gaccgttgtc cggacagctc tggttctgtt atagacacca

1681 ggaacagtga agaactcaga gcaaaaagtt catcaggaac caacacatct gaccaaaagc

1741 aaagcaaagc aaaggggact cagctcctaa gcaggcatga tcggaaggaa cccttagtcc

1801 tgcacagagg gctagagcat acaaacacaa gtgcctgtca taaaaaccca acctccactc

1861 tgatgtgatc acaggttcag tgaactagga aagtaaggct ttaaactaaa atgtgtagaa

1921 aacagcctca atggaaagac cctcattcca aggctcacaa tagagaaaag cgctttacta

1981 cttacaaggc tgtgcacata ctcacttgtt tggaaatcaa agtagaatct ctttcctttg

2041 aatgtacagc tacattacaa tcattgataa aatcaagtgc tgcttctaac tccatcagca

2101 atctctcata cagcccactt ctgtcagtaa acggtccaca gtctaccaca atctcacaag

2161 gctgagaagt gtatctgtcc aggaccacca agtcagtcgc cgtctcagcg tcactcctga

2221 gaattcgctc cagtttctga atcttctctt ccaattcctc tggatcacat ttcccattta

2281 aaatggaagc agttagtccc aaaattcgag ggcatgatgg acaactctca cagagcttca

2341 taatttcacg ataggggtgg tctagaatcg caagatgaca ctcatcaaac accaaaaggt

2401 taatgtctga cagtgataag taaccatttt tcaaaacagt caaggcgaca tagcaagtca

2461 taatgagaac ctggtgctta gtaaactctt ggctccatct ctcttttgtc caagatgcat

2521 ttacttctag gtctgagtac tctccaacct tgagatccga gtgagttctg acggctgaca

2581 cttgttgagc aacctggttt gcagagttga cgaggaacac ggtccttttt gcatgcgggt

2641 tgaggtcgcc cctgatctga tgggccagct ctttggtgag caggaccgcg atgaacgtct

2701 tccctgagcc agtgttcaag cacacaattg tgttatggtc cagagctgct tccagcagct

2761 caacctggta ttttcttggc gtataaatgt tatcatgaat tgcttcttgt tgccatggca

2821 gtccaaagaa aggacccatt ggtgaggaag caggggtcat gagctgcagg ccagcgtaat

2881 ctggaacatc gtatgggtat gccatgctga ggggctgcaa agcagggctt ttcaatcatc

2941 cagtgtttct ttcattgcat tttgttctag cacagcttac tacaaaaggg aaaaagaata

3001 ccattacaca accatgcagg gttaaaacaa aaggaagcac gggaacaaga gagacatcac

3061 actactgctc cctcaaggag cacgggaaca agagagacat cacactactg ctccctcaag

3121 cctttcaggt tcttcctatg gctgctgtgg cttctcttga gatctaagag gatggtttta

3181 aaagtcaagc agtctatacc caaccaggca atcctacagc tagaagctta tctgaagaat

3241 gaaccagaca actgcacaaa tacataaaca cattaatttg cattgttaca ggaaaataca

3301 aatatataca tacacacaca tatacacaca tacactcttt ttaagttctc taatgttatg

3361 ttgacaaaat gagccaacgt acaggcaggc attaaagatg caaacacttg agctggttat

3421 atcactttat taaaaggctg ggaacaggaa tgttttgctt aactgacaac ctataactgc

3481 acattaaaac tatcctcaga catatacatt tacaatcata cccaccccaa taaactaaac

3541 ccatgatatt ccttacatgg aaaactgtcc acgtcatcta agcaccctcc cattttcaga

3601 tactctctcc agaagtaaat ttcactatgt aagttggaaa aatagaaatc ttttaaaacc

3661 tttctgtgtg tatgcacgca tgtgtaggcc agtgtgtgca agtgtggggc cagaagtcag

3721 cagtgatgtt cttgtccagc tctctcactc tgtgaggcag gaactctccc tggaactgag

3781 ctcagcatca gtaacactgg ggctgcatag cactgccctg agttctcacc agagtggctg

3841 gaactactta caggacaccc tgaggcaagt cttgctcaca gaagaatcgg gtcagggatc

3901 caggtggcac ttttcgggga aatgtgcgcg gaacccctat ttgtttattt ttctaaatac

3961 attcaaatat gtatccgctc atgagacaat aaccctgata aatgcttcaa taatattgaa

4021 aaaggaagag tatgagtatt caacatttcc gtgtcgccct tattcccttt tttgcggcat

4081 tttgccttcc tgtttttgct cacccagaaa cgctggtgaa agtaaaagat gctgaagatc

4141 agttgggtgc acgagtgggt tacatcgaac tggatctcaa cagcggtaag atccttgaga

4201 gttttcgccc cgaagaacgt tttccaatga tgagcacttt taaagttctg ctatgtggcg

4261 cggtattatc ccgtattgac gccgggcaag agcaactcgg tcgccgcata cactattctc

4321 agaatgactt ggttgagtac tcaccagtca cagaaaagca tcttacggat ggcatgacag

4381 taagagaatt atgcagtgct gccataacca tgagtgataa cactgcggcc aacttacttc

4441 tgacaacgat cggaggaccg aaggagctaa ccgctttttt gcacaacatg ggggatcatg

4501 taactcgcct tgatcgttgg gaaccggagc tgaatgaagc cataccaaac gacgagcgtg

4561 acaccacgat gcctgtagca atggcaacaa cgttgcgcaa actattaact ggcgaactac

4621 ttactctagc ttcccggcaa caattaatag actggatgga ggcggataaa gttgcaggac

4681 cacttctgcg ctcggccctt ccggctggct ggtttattgc tgataaatct ggagccggtg

4741 agcgtgggtc tcgcggtatc attgcagcac tggggccaga tggtaagccc tcccgtatcg

4801 tagttatcta cacgacgggg agtcaggcaa ctatggatga acgaaataga cagatcgctg

4861 agataggtgc ctcactgatt aagcattggt aactgtcaga ccaagtttac tcatatatac

4921 tttagattga tttaaaactt catttttaat ttaaaaggat ctaggtgaag atcctttttg

4981 ataatctcat gaccaaaatc ccttaacgtg agttttcgtt ccactgagcg tcagaccccg

5041 tagaaaagat caaaggatct tcttgagatc ctttttttct gcgcgtaatc tgctgcttgc

5101 aaacaaaaaa accaccgcta ccagcggtgg tttgtttgcc ggatcaagag ctaccaactc

5161 tttttccgaa ggtaactggc ttcagcagag cgcagatacc aaatactgtt cttctagtgt

5221 agccgtagtt aggccaccac ttcaagaact ctgtagcacc gcctacatac ctcgctctgc

5281 taatcctgtt accagtggct gctgccagtg gcgataagtc gtgtcttacc gggttggact

5341 caagacgata gttaccggat aaggcgcagc ggtcgggctg aacggggggt tcgtgcacac

5401 agcccagctt ggagcgaacg acctacaccg aactgagata cctacagcgt gagctatgag

5461 aaagcgccac gcttcccgaa gggagaaagg cggacaggta tccggtaagc ggcagggtcg

5521 gaacaggaga gcgcacgagg gagcttccag ggggaaacgc ctggtatctt tatagtcctg

5581 tcgggtttcg ccacctctga cttgagcgtc gatttttgtg atgctcgtca ggggggcgga

5641 gcctatggaa aaacgccagc aacgcggcct ttttacggtt cctggccttt tgctggcctt

5701 ttgctcacat ggctcgacag atctgcgcag caccatggcc tgaaataacc tctgaaagag

5761 gaacttggtt aggtaccttc tgaggcggaa agaaccagct gtggaatgtg tgtcagttag

5821 ggtgtggaaa gtccccaggc tccccagcag gcagaagtat gcaaagcatg catctcaatt

5881 agtcagcaac caggtgtgga aagtccccag gctccccagc aggcagaagt atgcaaagca

5941 tgcatctcaa ttagtcagca accatagtcc cgcccctaac tccgcccatc ccgcccctaa

6001 ctccgcccag ttccgcccat tctccgcccc atggctgact aatttttttt atttatgcag

6061 aggccgaggc cgcctcggcc tctgagctat tccagaagta gtgaggaggc ttttttggag

6121 gcctaggctt ttgcaaaaag cttgattctt ctgacacaac agtctcgaac ttaagctgca

6181 gaagttggtc gtgaggcact gggcaggtaa gtatcaaggt tacaagacag gtttaaggag

6241 accaatagaa actgggcttg tcgagacaga gaagactctt gcgtttctga taggcaccta

6301 ttggtcttac tgacatccac tttgcctttc tctccacagg tgtccactcc cagttcaatt

6361 acagctctta aggctagagt acttaatacg actcactata ggctagacac catggtgtct

6421 aagggcgaag agctgattaa ggagaacatg cacatgaagc tgtacatgga gggcaccgtg

6481 aacaaccacc acttcaagtg cacatccgag ggcgaaggca agccctacga gggcacccag

6541 accatgagaa tcaaggtggt cgagggcggc cctctcccct tcgccttcga catcctggct

6601 accagcttca tgtacggcag cagaaccttc atcaaccaca cccagggcat ccccgatttc

6661 tttaagcagt ccttccctga gggcttcaca tg

//

**Supplementary Figures**

**S1 Fig. Analysis of ESC lines for *Dicer^SOM^* mouse model production by PCR and Western blotting.** The yellow marked ESC lines were used for producing chimeric mice, the clone 11 (G2) gave rise to *Dicer^SOM^* animals used in the experiment.

**S2 Fig. Additional micrograps documenting spindle defects in meiotically maturing DicerSOM/SOM oocytes**. Framed micrographs were used for Fig. 2A. Size bar = 10 µm.

**S3 Fig. Comparison of transcriptomes of oocytes affected by different mutations in RNAi pathway.** (A) Pairwise comparisons of relative gene expression changes using DESeq2-normalized expression values. In red are depicted genes potentially targeted by RNAi (i.e. with identified antisense siRNAs). (B) Correlation matrices calculated from RPKM values all annotated genes (upper panel) and predicted siRNA targets (lower panel).

**S4 Fig. Comparison of transcriptomes of oocytes affected by mutations of RNAi and/or piRNA pathway.** MA plots with the same Y-scale depict changes relative to controls (*Mili*^DAH/WT^, *Dicer*^SOM/WT^) and double mutant (*Mili*^DAH/DAH^, *Dicer*^SOM/SOM^) relative to RNAi mutant (*Mili*^DAH/WT^ *Dicer*^SOM/SOM^). Transcripts with significantly higher and lower transcript levels are shown in red and blue, respectively. Transcripts from a tandemly duplicated cluster at chromosome 12 are depicted as triangles.

**S5 Fig. Selected long but not necessarily intact L1 retrotransposons from selected abundant subfamilies with the best coverage in RNA-seq data from double mutant data.** Shown are UCSC genome browser snapshots of specific L1 insertions, their % coverage and the longest covered sequence by RNA-seq data in double mutants. The samples (from the top) depict merged data from 125 nucleotide paired-end (125PE) sequencing of normal full-grown GV oocytes from Horvat et al. [[54](#_ENREF_54)], 75 nucleotide paired-end (75PE) sequencing of controls (*Mili*^DAH/WT^, *Dicer*^SOM/WT^) and double mutants (*Mili*^DAH/DAH^, *Dicer*^SOM/SOM^) from this study, and 50 nucleotide single-end (50SE) sequencing of total transcriptome of 10 days old wild-type and *Mili*^-–/-–^testes[[28](#_ENREF_28)]. All RNA-seq data were scaled and are shown at the same scale 5 CPM indicated by the dashed line. The scheme below L1Md_A depicts intact ORF1 and ORF2 as lighter and darker grey rectangle, respectively.

**S6 Fig. Lack of positive staining for L1 ORF1 in fully-grown oocytes**. Oocytes were stained with α-L1-ORF1 antibody ([[73](#_ENREF_73)] generous gift from Donal O’ Carroll) and analyzed by confocal microscopy. Shown are 10 μm optical sections. L1 ORF1 signal is in red channel, DNA stained with DAPI is shown in blue color. Size bar = 30 μm.

**S7 Fig. Expression of piRNA pathway factors in mouse newborn ovaries lacking *Sohlh1* and *Sohlh2*.** RNA-seq data were obtained from a published dataset PRJNA293873 [[61](#_ENREF_61)] and gene expression was analyzed as described in Methods.

**Supplementary Tables**

**S1 Table. Differentially expressed genes in RNAi & piRNA mutant/RNAi mutant**

**S2 Table. Primer table**

**S3 Table. RepeatMasker retrotransposon groups**

**S4 Table. siRNA target list**

**S5 Table. Full-length L1 table**

**Supplementary Files**

**S1 File. Numerical data for graphs**
