# Supplementary figures and images for: Restricted and non-essential redundancy of RNAi and piRNA pathways in mouse oocytes
Source: PLoS Genet. 2019 Dec 20;15(12):e1008261. doi: 10.1371/journal.pgen.1008261 (PMC6944382; doi:10.1371/journal.pgen.1008261)

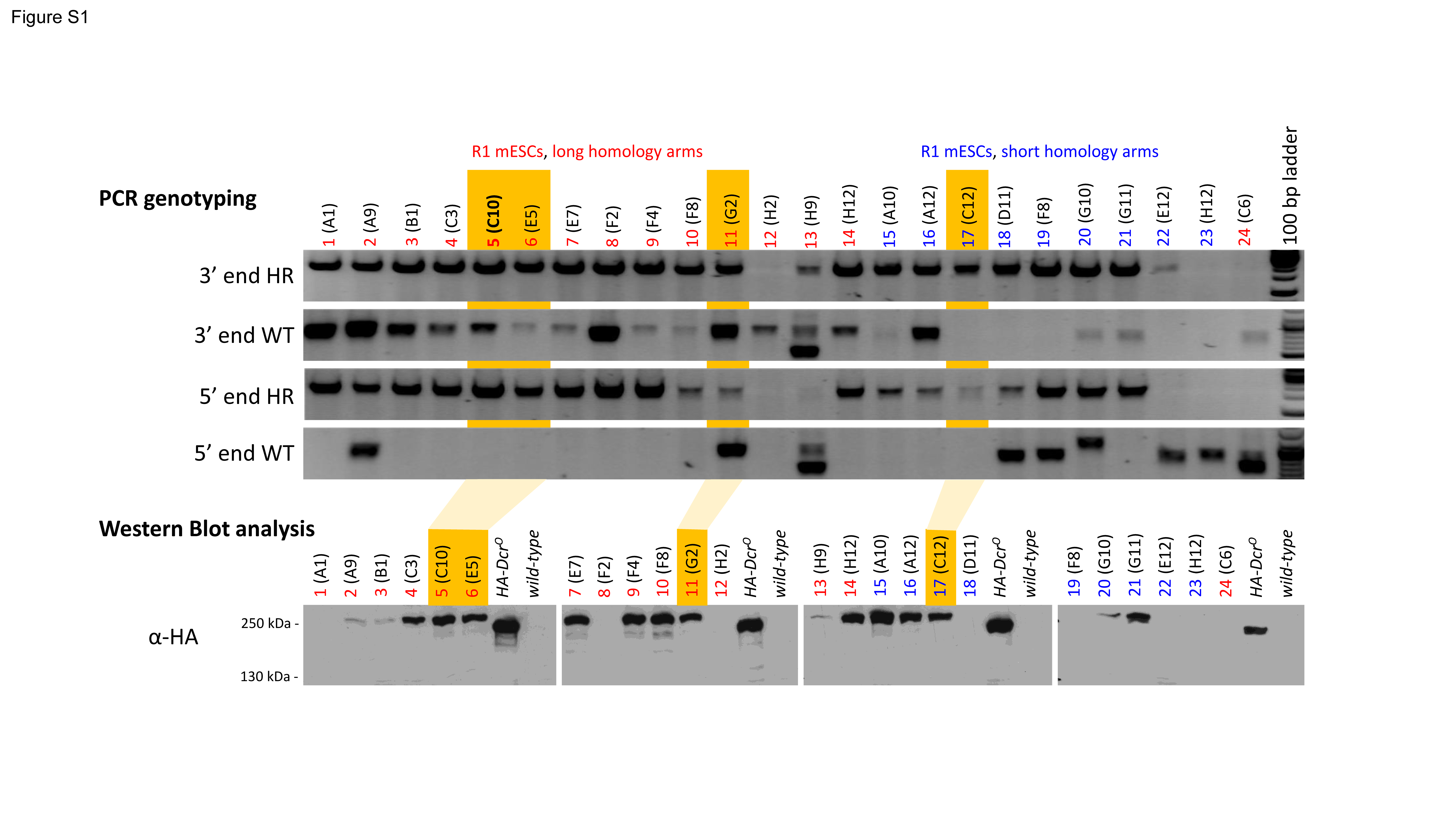

Supplement: S1 Fig — The yellow marked ESC lines were used for producing chimeric mice, the clone 11 (G2) gave rise to DicerSOM animals used in the experiment. (TIFF) [file pgen.1008261.s002.tiff]

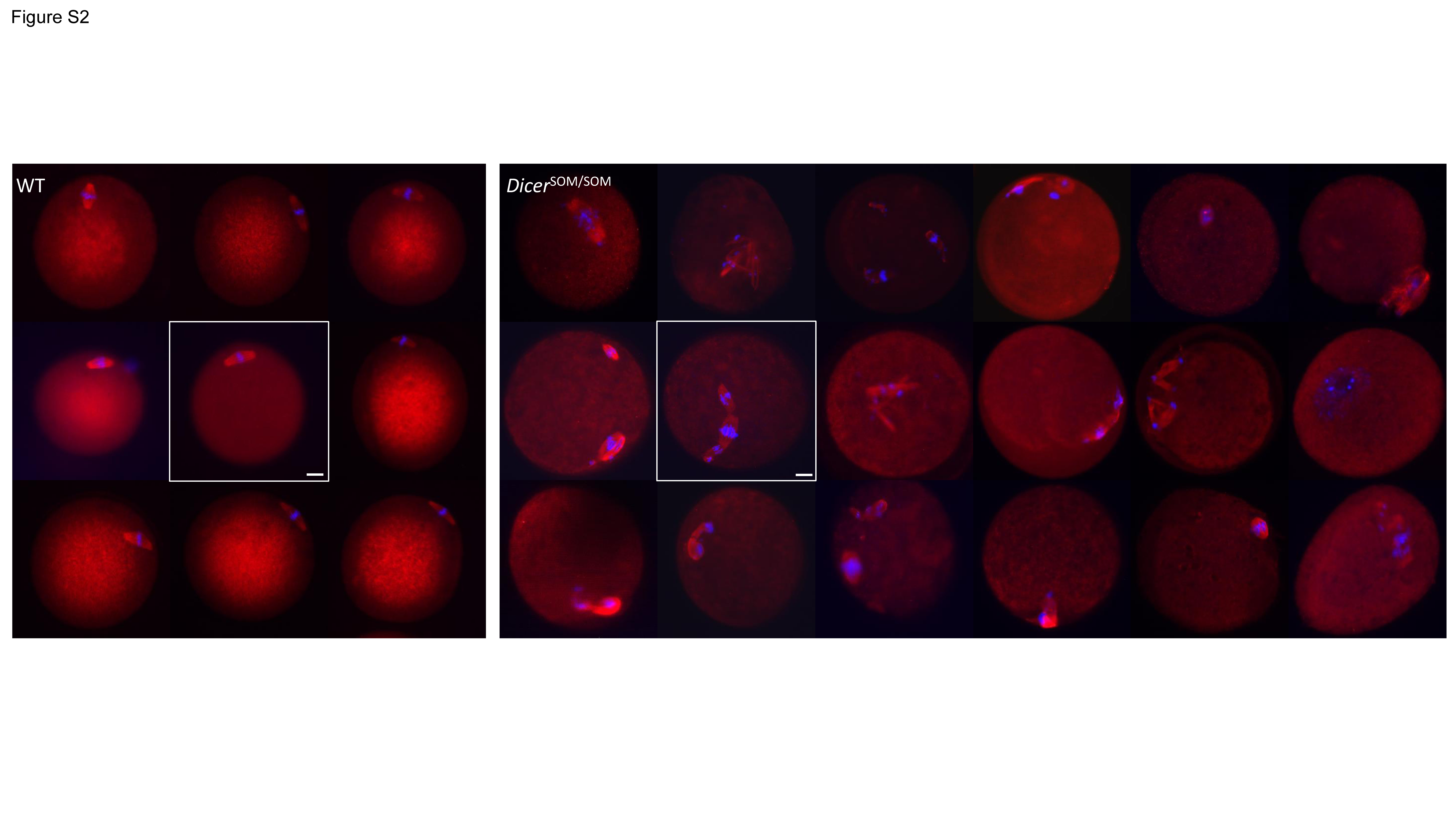

Supplement: S2 Fig — Framed micrographs were used for Fig 2A. Size bar = 10 μm. (TIFF) [file pgen.1008261.s003.tiff]

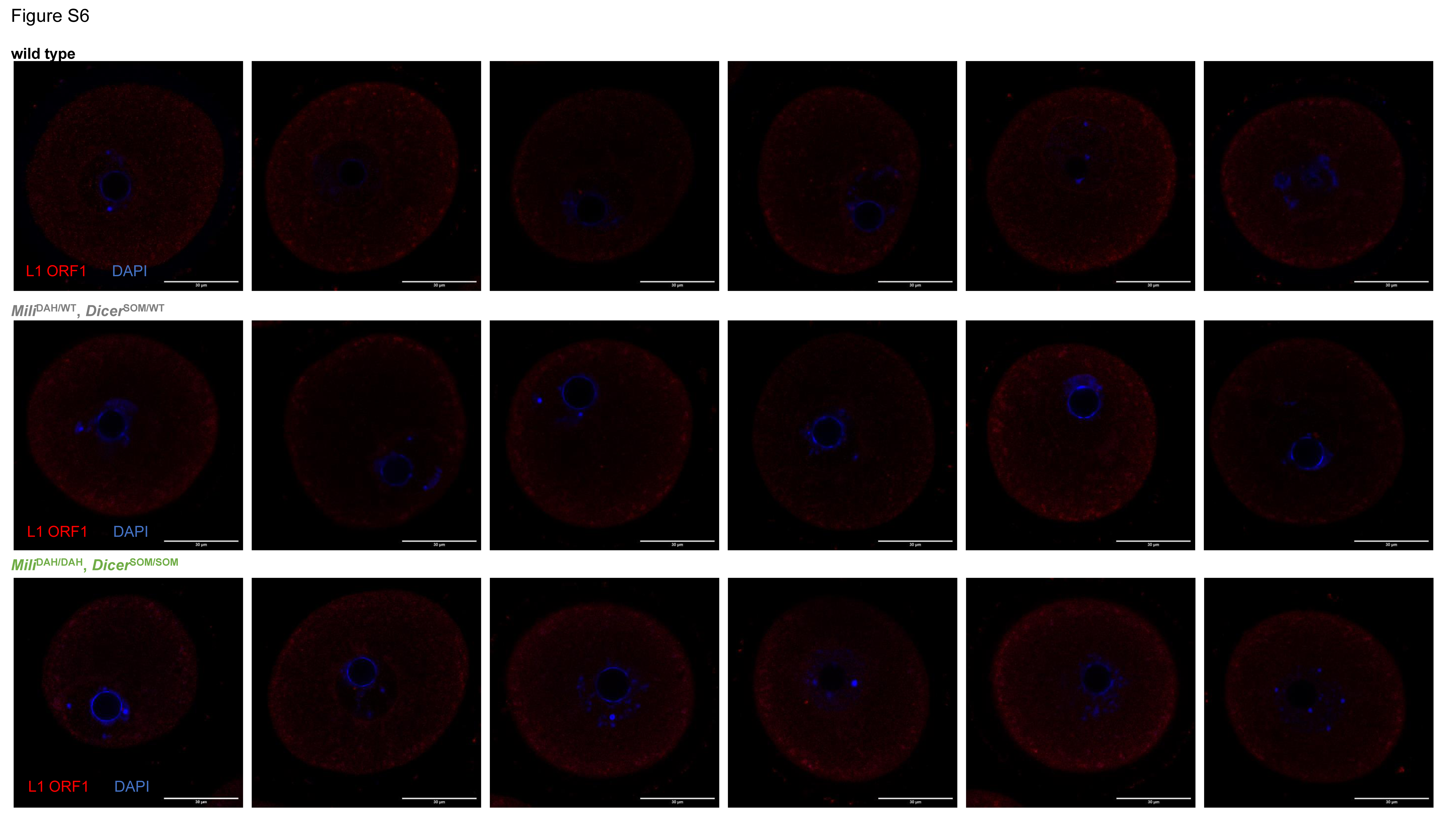

Supplement: S6 Fig — Oocytes were stained with α-L1-ORF1 antibody ([73] generous gift from Donal O’ Carroll) and analyzed by confocal microscopy. Shown are 10 μm optical sections. L1 ORF1 signal is in red channel, DNA stained with DAPI is shown in blue color. Size bar = 30 μm. (TIFF) [file pgen.1008261.s007.tiff]
