# Supplementary material for: Restricted and non-essential redundancy of RNAi and piRNA pathways in mouse oocytes
Source: PLoS Genet. 2019 Dec 20;15(12):e1008261. doi: 10.1371/journal.pgen.1008261 (PMC6944382; doi:10.1371/journal.pgen.1008261)

Reviewer 1:

Major comments:

1. Small RNAseq data in figure one is in from Yang et al., 2016. Small RNAseq data should be acquired for single and double mutants to validate putative RNAi targets from mRNA seq, as well as show changes in global production of siRNA and piRNA with mutations.

Ad validation of putative RNAi targets: We are convinced that an additional small RNA-seq is not necessary for validating putative RNAi targets as the targets were validated using small RNA-seq data already. For generating the putative RNAi target list, which we have used in the manuscript (supplementary file 2), we combined mapping of three referenced independent small RNA datasets (10.1038/nature06904, 10.1126/sciadv.1501482, 10.1261/rna.048215.114) and our *de novo* assembly and annotation of lncRNAs published in Karlic et al., 2017 (10.1093/dnares/dsw058). We manually inspected ~500 endogenous genes to which mapped the highest amount of antisense small RNAs and identified their source - antisense pseudogene-carrying lncRNAs, convergent transcription, or inverted repeats. The supplementary file 2 contains 197 protein-coding genes with the highest number of antisense-siRNAs with average RPM abundance >50. There is still more than 300 genes for which exist *bona fide* endo-siRNA substrates but are targeted by lower amounts of endosiRNAs than the targets we used. The fact that only a fraction of the targets shows strong upregulation in Dicer mutants likely reflects small RNA:target stoichiometry and constrained RNAi activity because of restricted expression of maternal AGO2, the effector of the pathway (10.1016/j.cub.2017.11.067).

Ad impact of mutations on small RNAs: We agree that it would be nice to have a detailed overview of changes of small RNAs in mutants in addition to retrotransposon expression analysis but this would not significantly change the main outcomes of our work – (i) absence of an additional phenotype in RNAi & piRNA pathway double mutants, (ii) different targeting of different retrotransposons by RNAi and piRNA pathways, and (iii) minimal expression of L1 in oocytes. We would like to point out that the key element of our work are genetic mutations of key components of RNAi and piRNA pathways and we aimed at characterizing effect of these mutations on fertility and expression of retrotransposons, not on mechanistical details of how defective siRNA & piRNA biogenesis would impact small RNA population, which can be expected to undergo complex remodeling. We have qPCR data (as we reported for the MT mutants) showing that miRNA abundance is upregulated in the SOM mutant, suggesting that siRNAs produced from long dsRNA are being replaced by miRNAs - but this is a subject of another story and beyond the scope of this work.

The reason we did not produce small RNA-seq is that: (1) we do not have a functional protocol, which could be used for small amount of material and (2) the limited amount of experimental animals. We would like to point out that small RNA-seq in oocyte does not have a standardized routine protocol. We tested two commercial kits for small RNA-seq from small input but the result were not satisfactory. One of the contributing issues possibly was that miRNA abundance (and small RNA abundance in oocytes in general) is low – an oocyte actually has the same amount of miRNA molecules as a somatic cell. This seems to complicate the use of commercial kits designed for small amounts of total RNA from somatic cells because the oocyte has ~50x less small RNAs per total RNA (https://doi.org/10.1101/757153). Previous sequencing protocols for small RNAs from mammalian oocytes required large amount of input material except of pioneering work of Q. Wang et al., whose earlier data we used (10.1126/sciadv.1501482) and who published a single-cell protocol at the end of July 2019 (https://doi.org/10.1038/s41467-019-11312-8). This single-cell protocol is rather complex and our lab does not have resources to establish this protocol and use it for sequencing double mutants. The previous protocol for small amounts by Wang et al. was based on isolating ~100 ng of total RNA and sequencing a fraction of it. 100 ng of total RNA could be isolated from over

200 oocytes = 8-10 females, which makes it not realistic for sequencing RNAi & piRNA double knock-out even in a duplicate within a reasonable revision time frame.

2. Aside from eliminating truncated Dicer variant, the Dicer SOM mouse doesn't seem to significantly change the overall phenotype compared to Dicer deltaMT, except from few putative RNAi targets and upregulated genes in RNA-seq. Not really significant or novel that this mouse was generated.

We disagree with this interpretation:

1) Dicer$^{\Delta MT}$ animals still express a truncated Dicer isoform (10.1101/gr.216150.116) because of a second alternative promoter. Because of that, their oocytes exhibit residual RNAi, which manifests as weaker upregulation of genes regulated by endogenous RNAi and subfertility – Dicer$^{\Delta MT}$ females occasionally produce a progeny. Thus, Dicer^{SOM}, which lacks the entire intron 6, is superior to Dicer$^{\Delta MT}$ in terms of the strength of the phenotype, i.e. suppression of endogenous RNAi.

2) Dicer^{SOM} model has eliminated introns 2-6 – it thus demonstrates that introns 2-6 are non-essential for development and male fertility. This makes is an excellent control for precise dissection of the phenotype of mice lacking the N-terminal helicase domain.

3) Dicer^{SOM} stands out from options for studying loss of RNAi in mouse oocytes because Dicer$^{\Delta MT}$ has a weaker phenotype while conditional mutagenesis is labor-intensive, would involve many more animals and could be problematic because Cre-recombinase expression drivers (promoters) may induce Cre-expression too early or too late (i.e. post-meiotic Cre-drivers co-expressed with short Dicer variant mays suppress RNAi too-late while pre-meiotic Cre-drivers may affect the miRNA pathway).

4) Dicer^{SOM} model excellent for studying miRNA pathway inefficiency in oocytes. Conditional Dicer deletion eliminates all Dicer-dependent small RNAs including miRNAs while in Dicer^{SOM} model, RNAi is suppressed similarly to the conditional Dicer knock-out but miRNAs are not lost because of the residual full-length HA-tagged Dicer isoform. miRNA levels actually increase (replacing missing endo-siRNAs), offering a unique model for boosting endogenous maternal miRNA function *in vivo*. Dicer^{SOM} offers better increase in miRNA levels because Dicer$^{\Delta MT}$ model is still expressing low levels of truncated Dicer. We recently showed that miRNA levels in oocytes are too low to mediate reporter repression while three-fold increase in miRNA amount per oocytes restores miRNA repression (fig. 4A - doi.org/10.1101/757153). A similar increase in miRNA abundance is observed in Dicer^{SOM}, which makes it an excellent model to study miRNA pathway in growing and fully-grown oocytes.

5) Dicer^{SOM} carries an HA tag at the N-terminus, which allows for tracing full-length Dicer expression. An HA-tag Dicer knock-in was generated (10.1371/journal.pgen.1006095). While our model is not novel in this sense, it allows for testing reproducibility of the reported patterns and can be used for analyzing Dicer expression with easily accessible anti-HA antibodies. This will be important for complementing the aforementioned mouse model lacking the N-terminal helicase domain (made simultaneously with Dicer^{SOM}, just using a different recombination cassette).

3. What is a "genomic checkpoint" that the piRNA pathway relies on? Describe better as this is mentioned twice.

"Genomic checkpoint" refers to a genomic locus generating a primary piRNA transcript – it functions as a checkpoint detecting active transposable elements: once a transposable elements enters the checkpoint (i.e. inserts into the locus and becomes a part of the piRNA transcript), it will be recognized and silenced. We revised the text to make this point clearer.

4. Explain better the significance of including comparisons to *Ago2*, and differences in *Dicer* vs *Ago2* oocyte phenotypes?

We included *Ago2* data, which were part of Stein et al. 2016 dataset published in PLOS Genetics (doi.org/10.1371/journal.pgen.1005013) because they represent yet another means of suppression of RNAi, each of which could have slightly different impact on the maternal transcriptome because the four mutants (*Dicer^{SOM}*, *Dicer$^{-/-}$*, *Ago2$^{m/m}$*, and *Ago2$^{-/-}$* would have different impact on composition of small RNA populations:

- *Dicer$^{SOM/SOM}$* oocytes lack the main siRNA-producing activity while miRNAs remain produced and loaded onto all AGO proteins.
- *Dicer$^{-/}$* oocytes lack all Dicer-dependent small RNAs but AGO proteins remain there, presumably being loaded with Dicer-independent small RNA.
- *Ago2$^{m/m}$* oocytes have intact biogenesis of small RNAs but lack ability to cleave small RNA targets.
- *Ago2$^{-/-}$* oocytes lack not only the cleaving activity but the entire protein, which should result in smaller small RNA pool in the oocyte (reduced by the number of small RNAs, which would be loaded onto AGO2). This could explain separate clustering of Ago2 mutants from Dicer in Stein et al. 2015 data.

Our correlations primarily show that despite different means of suppression of RNAi (and the apparent bench effect manifested on PC1), transcriptome changes in Dicer^{SOM} mutants are largely reproducing changes in Stein's RNAi pathway mutants and are better than those in Dicer$^{\Delta MT}$. To address this comment, we revised the result section to highlight differences among the mutants.

Minor comments:

Figure 2B. Missing a loading control.

In Fig. 2B we used equal loading of 40 µg of lysate upon quantifying the protein amount by the Bradford assay. We did not use a specific loading control such as tubulin because of diversity of the tissues. In addition, we preferred to show specificity of HA signal by showing a larger part of the Western blot. However, we have available Ponceau S staining of the Western blot membrane. To address reviewer's comment, we inserted the Ponceau S staining into the revised version.

Figure 2C. Needs labels, what is green and blue? What is the genotype?

In Fig. 2C, Dicer was shown in green, blue was DAPI staining of DNA. We added labels and genotype and show a new image as reviewer 2 requested an image of better quality. We now show a split image of a seminiferous tubule from wild type animals and Dicer^{SOM} homozygotes. Dicer (i.e. α-HA antibody staining) is now shown in red.

Figure 2E. Endogenous control gene for qPCR?

We used *Hprt1* as an endogenous control for qPCR , but we also run in parallel Beta-2-microglobulin (*B2m*) to make sure normalization to *Hprt1* would not introduce a major bias.

Figure 3B. n oocytes are from how many females?

It was eight females with each genotype (two independent experiments with four females). This information was added to the text. The relatively low numbers of oocytes were mainly caused by losses during isolation, preparation for staining, and challenging localization of stained oocytes on slides.

Figure 3C. Reference for initial set of genes that are sensitive to RNAi pathway?

As described above and in methods, the list of endogenous RNAi targets was produced by us by identifying most common gene-targeting antisense siRNAs and confirming their origin. Therefore, there is no reference to the initial set of genes that are sensitive to RNAi pathway. To be precise, this

is the list of ~200 genes, which are the most targeted by endogenous antisense siRNAs. There is many more genes for which exist antisense siRNAs while only a fraction of RNAi targets actually shows strong sensitivity to RNAi. However, it is not the point of the reviewed manuscript to discuss how functional endogenous RNAi integrates evolutionary history of antisense siRNA substrates and stoichiometry of siRNAs and their targets.

We revised description of the method section describing construction of the list, which reads now:

The potential siRNA targets (Supplementary file 2) were manually curated using the small RNA-seq datasets [32, 41, 43] and selecting annotated protein-coding genes for which existed antisense endogenous 21-23 nt small RNAs with > 50 RPM average abundance and for which we could identify their origin.

Figure 3D. Need to validate putative RNAi targets using small RNAseq. Otherwise gene expression differences could be indirect and due to oocyte phenotypes.

Given the origin of the list described in detail in the response to the point 1 and above, we believe we do not need to validate putative RNAi targets using small RNA-seq. Three different RNA-seqs were used to identify targeted genes and the source of the targeting siRNAs was identified. Importantly, the list was produced independently of gene expression changes because it is the list of top 197 genes with >50 RPM antisense siRNA mapping to them where we could validate the origin of those antisense siRNAs. We opted for this construction of the list to avoid precisely the issue pointed out by the reviewer: selection based on gene expression differences could be misleading because of the complexity of transcriptome changes in Dicer mutants would yield false positive and false negative results.

Figure 3E. What is the difference between Ago2 mutants from Stein et al., 2015 in orange vs. pink? Triangle vs. square symbols?

Orange squares represent catalytically dead mutant *Ago2*$^{m/m}$, pink squares knock-*out Ago2*$^{-/-}$. We revised the figure to indicate this fact directly in the panel 3E.

Figure 4A. More detailed quantification of primordial follicles vs advanced follicles, maybe there is a difference at that resolution between WT and mutants.

In the revised version, we add quantitative analysis of histological sections, which could be obtained and analyzed. It did not reveal an apparent significant difference (new Fig. 4D).

Figure 4D. Better to use RNA-seq to analyze abundance of TEs in mutants to eliminate background elements within genes that are changing as a consequence of mutation.

Although it is questionable whether RNA-seq analysis would eliminate background elements as there would be still multimapping reads, which could come from elements within genes, we agree that RNA-seq offers an independent mean for analysis of retrotransposons and would be more comprehensive. We therefore performed RNA-seq analysis of mutants during revision and added it as a new figure (new Fig. 5). RNA-seq data were added to the previous GEO submission GSE132121.

Also, how many biological replicates per bar in qRT-PCR?

For qPCR, we used three biological replicates (i.e. oocytes from different animals) per bar.

Reviewer #2:

Minor comments

1. The quality of images used in Figure 2C is poor. Its legend lacks important information such as which panel shows what with which antibody.

We produced new images of seminiferous tubules and added the requested information.

2. The quality of images used in Figure 3A is also poor.

Image 3A was obtained using a regular fluorescent microscope as it was sufficient to observe spindle defects and was easier to use than scanning confocal microscopy. We agree that it would be nicer to have clear hi-res confocal images of defective spindles but we believe that the current figure 3A nonetheless illustrates the major difference between spindle structure in WT and Dicer$^{SOM/SOM}$ maturing oocytes. In fact, even omission of spindle images would not change conclusions of the work. Below we provide additional images documenting the major difference between spindle architecture in WT and Dicer$^{SOM/SOM}$ oocytes, white frames depict those used in the manuscript.

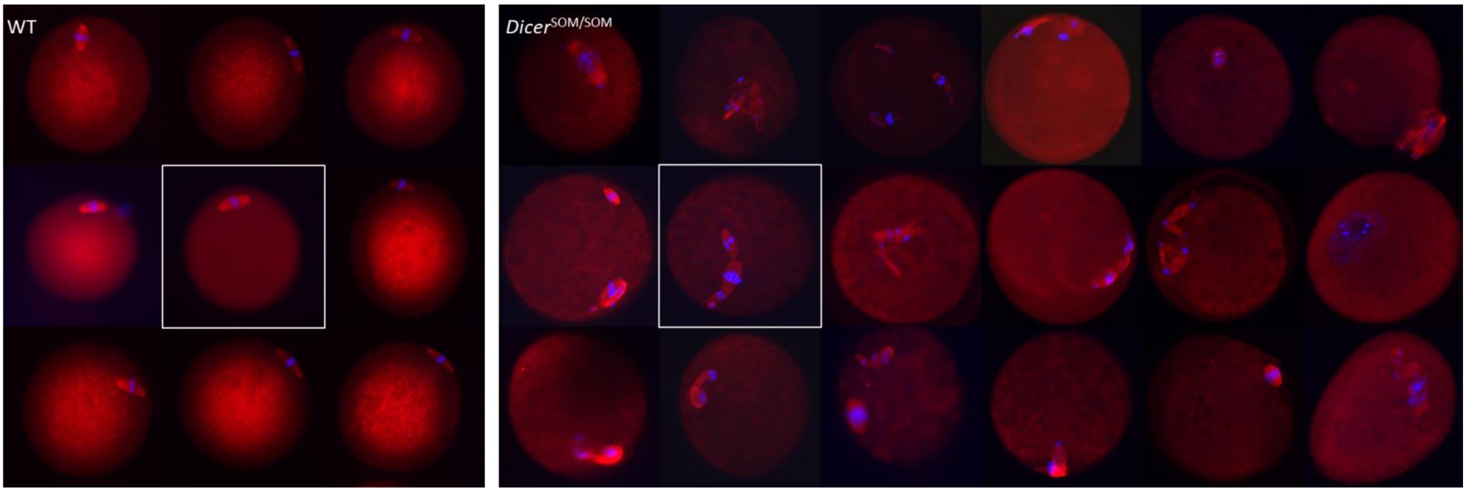

3. Figure 3C: The authors should add non-targeted genes in this experiment.

The analysis included two non-targeted "housekeeping" genes – *Hprt1* and *B2m*. *Hprt1* was used for calculating relative expression, *B2m* was added to the graph.

4. Figure 4A – C: here the authors analyze ovarian weight, histology and number of fully-grown GV oocytes using mice with age between 8 and 18 weeks. The authors should describe in legends the age in weeks of mice they use for each experiment.

For ovarian weight, histology, fully-grown oocyte yield, and follicular quantification, animal age was added to figure legends and animal age is also given in tables accompanying graph data.

In my opinion, the authors should use mice with same age in weeks in these experiments.

We reviewed available data and tried to narrow down the age from 8-18 weeks. For example, for ovarian weight, we collected data from 14-19 weeks old animals, and for oocyte yield it is seven 10-16 weeks old females (we could also use five 13-16 weeks old, the result would be the same – no significant difference).

In addition, the authors' better present data with male mice in Figure 4D for the reader to understand the degree of contribution of RNAi and piRNA pathways to suppress TEs in female gonads.

In the revised manuscript, we provide RNA-seq data from single and double mutant oocytes, which complement the previous qPCR analysis and well demonstrate the degree of contribution of RNAi and piRNA pathways to suppression of different types of TEs. Direct comparison oocyte data with spermatogenesis is difficult because a published relevant P10 Mili$^{-/-}$transcriptome (Manakov et al., Cell Rep. 2015, GSE70731) comes from the whole testis (i.e. transcriptome changes in primary spermatocytes would be diluted in the total testis transcriptome) and was performed as 50SE (less stringent mapping). However, we decided to include these data into Fig. 6 expression profiles of individual L1 elements despite these caveats (which are discussed) as we thought it is better to show these data normalized the same way side by side with oocyte data than not doing that.

Reviewer #3:

The upregulation of RNAi targets in figure 4D & F could also be possibly presented in cumulative frequency plots (S-curves). These have traditionally been used for miRNA effects and are quantitative and very easy to understand.

We also generated S-curves (see below). However, we are not sure whether transformation of scatterplots to S-curves would be more informative and would make better data representation than the used MA-plots and scatterplots because S-curves do not reveal transcript abundance.

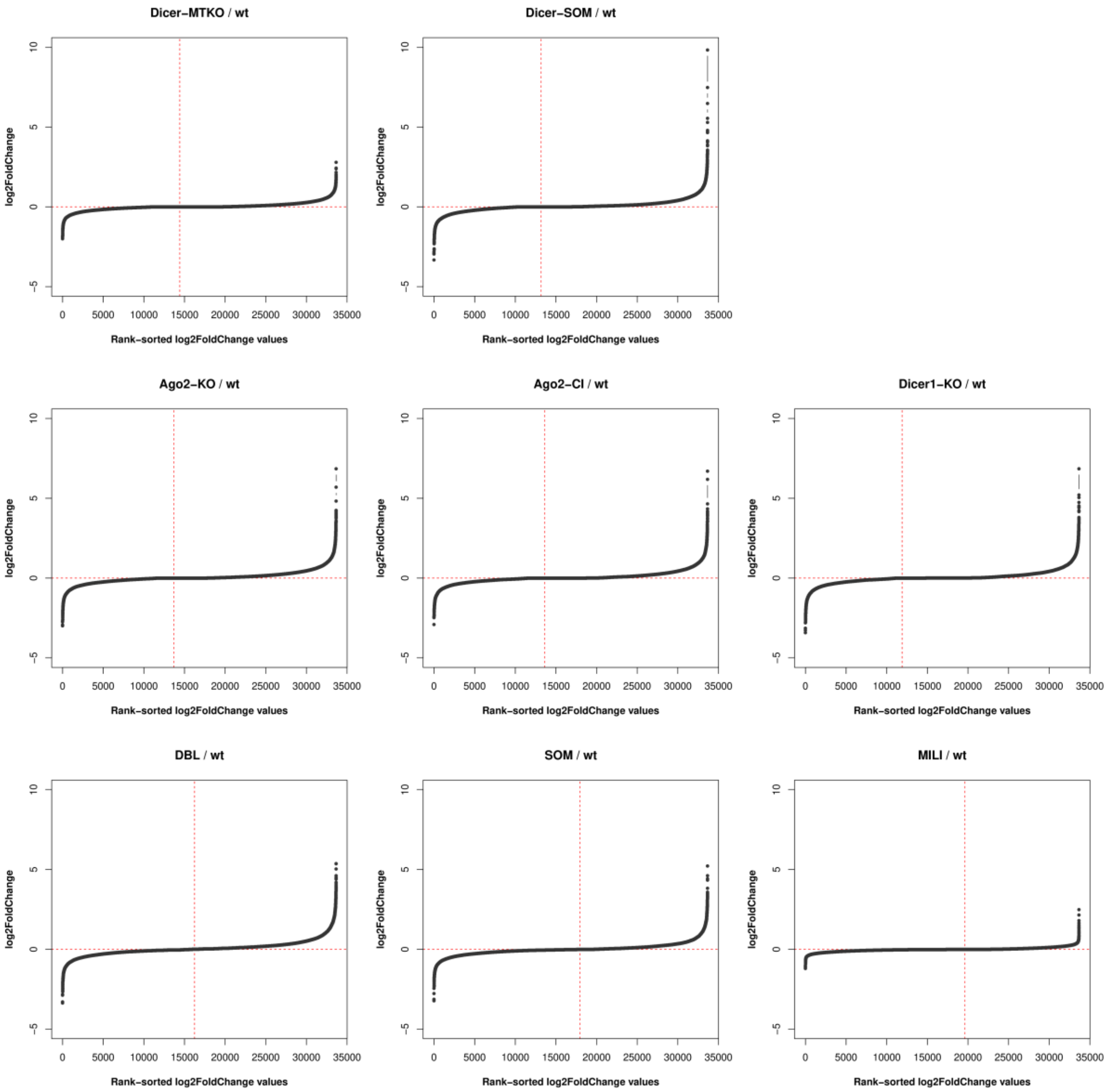

The data presented in Figure 5 is not presented in the results section. This needs to be rectified.

We used these data in the discussion and not results as this was re-analysis of published data done for the purpose of discussion. With the new RNA-seq data that we generated for the revised version, we include the last figure into results as Fig. 6. Please note that the current Figure 6 used different mapping strategy with a higher stringency and that L1_Md_F2, _Gf, and _T patterns in Fig. 6C are highly similar but not identical, presumably due to reads mapping to their consensus sequence.

The data presented in Figure 5 is an extremely important observation. I think it should be given some mention in the abstract.

We revised the abstract to address the comment (the last two sentences) to address this point.

I also think the key message from the low levels of L1 transcription in the female germline and minor upregulation of L1 transcripts (compared to the male germline) in the absence of the RNAi and/or piRNA pathways would be substantiated by staining for L1 ORF1 protein. The expectation would be that it should not be detected in the respective mutant genotypes.

We examined L1 ORF1 expression by staining with antibody generated by Donal O'Carroll's group but we could not detect its signal in oocytes (WT and double mutants while the antibody generates clear signal mutant testes). The negative staining results was included to the supplementary data if it would be desirable to have it as a part of the work.

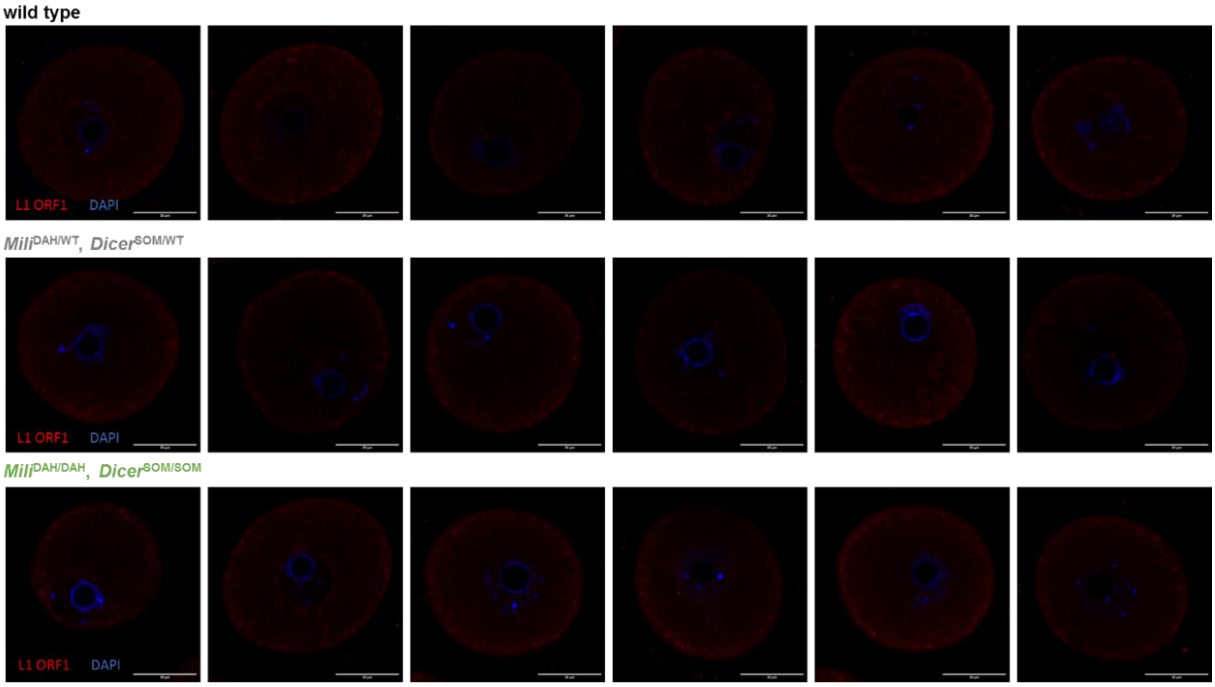

I think the discussion should give a sentence or two on why the piRNA-pathway expression in should be retained in the female germline of species that does abundantly express active TEs therein. The piRNA pathway is more than just the PIWI protein but requires the expression of many biogenesis factors (at least >10). Could the expression of the pathway be an insurance policy against a future invasion?

We revised discussion to address reviewers point. One of the factors likely contributing to retention of the piRNA pathway in the female germline could be transcriptional control common for both germlines. We examined expression of piRNA pathway components in oocytes lacking *Sohlh1/2* transcription factors, which are also expressed during spermatogenesis (10.1172/JCI90281) and indeed, there was a clear reduction of expression of many of the components ->.

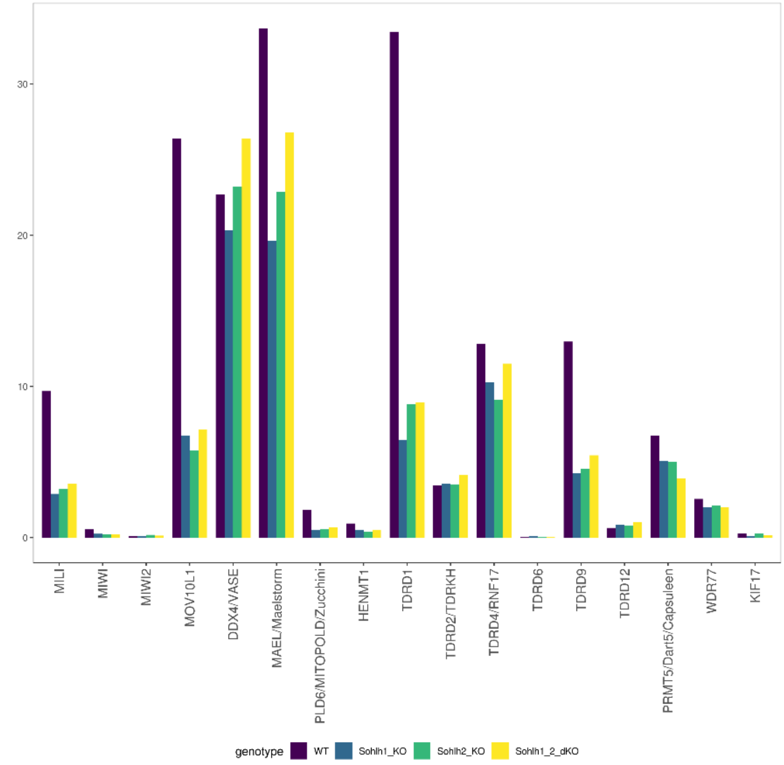

Supplement: Attachment — Submitted filename: 2019 RNAi & piRNA reviewer response.pdf [file pgen.1008261.s015.pdf]
