# Supplementary material for: Restricted and non-essential redundancy of RNAi and piRNA pathways in mouse oocytes
Source: PLoS Genet. 2019 Dec 20;15(12):e1008261. doi: 10.1371/journal.pgen.1008261 (PMC6944382; doi:10.1371/journal.pgen.1008261)
Supplement: Attachment — Submitted filename: 2019 RNAi & piRNA reviewer response 191121.pdf [file pgen.1008261.s016.pdf]

Reviewer 1:

Reviewer #1: Overall, I am satisfied with authors' responses.

I still have an issue with the genomic "checkpoint". The current draft describes piRNA clusters as "... specific genomic regions serving as "checkpoints" sensing invading mobile elements". This is not entirely correct since a genomic region senses nothing by itself. If not expressed, its resident sequences are invisible to the piRNA system and the cell. It is an unnecessary invention that will only confuse readers, and I ask authors to refrain from using this term. For example, the same idea can be expressed as follows:

"The piRNA pathway relies on specific genomic regions (piRNA clusters) whose expression primes piRNA production".

We opted for a different version, which would bring up the key concept we wanted to highlight using the criticized checkpoint analogy - that specific genomic regions serve as control points for sensing active mobile elements in the genome. We meant the checkpoint analogy in terms of a police/military strategy for protecting a large area where the enemy is screened and recognized at checkpoints. Furthermore, like a police or military checkpoint is a physical place serving its control function when it is manned, a piRNA cluster region is a genomic "checkpoint" region functional only in the germline when piRNA factors are present. A piRNA cluster region is visible to the piRNA system because it is a part of it. A piRNA cluster, checkpoint for active mobile elements, it is invisible to the cell without the piRNA system – like in somatic cells not expressing piRNA factors. We thus do not think the analogy is an "unnecessary invention" as it helps to understand how piRNA system works, evolves, and differs from RNAi-based recognition and silencing of mobile elements.

We recognize that analogies have limits, this is their nature. We also recognize that the term checkpoint is being mainly used in biology in the context of quality control and gate-keeping in cell cycle and DNA damage research and that this could be a source of confusion, although we do not think our text could be confused with that other meaning, particularly since we put "checkpoint" into quotation marks. In any case, to address reviewer's comment we use the term sensor instead of the checkpoint and revised the two instances of "checkpoint the following way:

page 3 (Author Summary)

The piRNA pathway is an ancient germline defense system analogous to acquired immunity: once a retrotransposon jumps into a piRNA-producing locus, which provides a kind of a "genomic sensor" for actively transposing elements, it is recognized and suppressed.

page 4 (Introduction)

The piRNA pathway relies on specific genomic regions (piRNA clusters) that are sensing invading mobile elements and giving rise to piRNAs. These are 25-30 nucleotides long RNAs loaded onto PIWI subgroup of the Argonaute protein family, which guide retrotransposon recognition and repression [15].

Reviewer #2

On the whole it has been improved and the authors have addressed some of the reviewers' concerns. However, it is already known that the deficiency of PIWI genes results in no discernible phenotype in female mice. In contrast, the authors previously found that the deficiency of the oocyte-specific Dicer variant causes frequent meiotic spindle defects upon resumption of meiosis. Thus, it is still not clear the rationale for why the authors try to examine possible redundancy between RNAi and piRNA pathways in mouse oocytes. The authors had better focus on characterization of Dicersom/som mutant oocytes in my opinion.

As stated in the introduction, we tested a hypothesis that highly active RNAi in mouse oocytes compensates loss of piRNA pathway, resulting in apparent lack of phenotype in PIWI knockout females:

It is unknown what accounts for the strikingly different phenotypes of piRNA pathway mutants in murine male and female germlines. In contrast to mice, Drosophila or zebrafish females lacking piRNA pathway components are sterile [17-19, 30]. It was hypothesized that the loss of the piRNA pathway in the mouse female germline could be compensated by RNA interference (RNAi) [31, 32].

Unlike continuously produced male germ cells, female germ cells enter meiosis before birth and become arrested in the prophase of meiosis I. They also undergo reduction in numbers, which has been proposed to be associated with activity of mobile elements, particularly L1 (ref 64: doi: 10.1016/j.devcel.2014.04.027). At the same time, the meiotic spindle defect is a post-ovulation phenotype observed in oocytes of adult animals defective in RNAi while RNAi is likely present during the entire oocyte development (given oocyte-specific expression of the short Dicer variant). Because small RNAs from oocytes suggested that both small RNA pathways retrotransposons simultaneously, it was possible that the loss of the piRNA pathway shows no phenotype in ovarian oocytes because it is rescued by redundantly acting RNAi pathway and vice versa. The redundancy would be limited to retrotransposon targeting while the unique role of endogenous RNAi in mouse oocytes manifests as the spindle defect phenotype, which appears upon ovulation. We believe that analysis of piRNA and RNAi pathway redundancy in retrotransposon repression in oocytes is interesting on its own given that both pathways are based on small RNAs but the mechanism of small RNA biogenesis is different as is how retrotransposons generate substrates entering both pathways.

Other points:

1. Figure 2 E & F show that expression levels of the full-length Dicer are markedly affected in Dicersom/som mutant oocytes. Why?

Our working hypothesis is that both MTC and MTA LTRs, which function as oocyte specific promoters (and perhaps additional MaLR LTRs in intron 6 as well), have a positive effect on the promoter of the full-length Dicer, i.e. act as its enhancers. We are trying to identify the specific transcription factors binding MT-elements in Dicer but we still do not have conclusive data. In any case, reduced levels of the full-length Dicer in oocytes should result in even closer Dicer knock-out phenocopy although the full-length Dicer is not efficiently producing siRNAs from long dsRNA substrates in mammalian cells (doi: 10.26508/lsa.201800289).

2. Figure 3A: the authors should show the figures provided to response to Reviewer #2 comments as supplementary figures.

The spindle defect image collection was included as Fig. S2.

3. The figure showing L1 ORF1 staining in the authors response to reviewer #3 appears to show that part of chromosomes (DAPI staining) is already excluded from the nucleus in Dicer mutants. This may imply that the deficiency of the Dicer activity already causes chromosome abnormality in early oocyte development and thus, the spindle defects observed are secondary.

Nuclear transplantation experiments showed that the sterility and the spindle defect are derived from oocyte’s cytoplasm (https://doi.org/10.1530/REP-08-0475). Furthermore, in our opinion, the presented confocal DAPI signal in ORF1 staining in surrounded nucleolus (SN) oocytes does not constitute solid evidence for chromosomal DNA excluded from the nucleus. In DAPI/ORF1 stainings where the nuclear/cytoplasmic boundary is well visible, the DAPI signal is rather localized at the nuclear periphery, which would be consistent with heterochromatin attachment to the nuclear lamina. During formation of SN chromatin configuration, chromatin condensates and aggregates and may appear as s a single isolated focus on an confocal image. Some signal deformation can also occur during oocyte mounting.

4. The figure showing expression levels of piRNA pathway genes in oocytes contains very important information in the field. Thus, the authors should show the figure in the current manuscript. Studies of this sort are good references and resources for further comparisons.

We placed the figure into the supplement (Fig. S7) as it makes a secondary discussion point derived from published data.

Reviewer #3:

All my concerns have been addressed.
